# The Common Task

**Published:** 1907-07-06

**Authors:** 


### THE COMMON TASK.

**Correspondence and Queries** for this section should be sent to the Editor of THE HOSPITAL, 28 Southampton Street, Strand, London, and marked "Nursing Administration."

### Low Contracts.

An acute hospital secretary was heard to murmur the other day, "This is what comes of cutting prices. The Board insisted on accepting the lowest contract for painting the walls, and now the builder is using Russian turpentine; I can smell it in the air." How many people responsible for the annual cleaning of institutions are able to detect the odour of Russian turpentine, or are acquainted with the reason why it ought not to be used?

### For the Glory of God.

The chapel is a familiar feature in most hospitals of any size, but it does not always take its proper place in the life of the household. Too often there are but two or three dullish Sunday services at which the staff reluctantly put in an appearance, greatly preferring the freedom from every-day associations which they can get in a church outside the walls. When it fulfils its highest function the chapel sets the tone of the household, and helps to dignify the commonest tasks. The ten-minute service at St. George's, held before the nurses go on duty in the morning, is an admirable example of what can be done even under difficult circumstances to sustain the highest aspects of the work; for the Nurses' Home is over half a mile from the hospital, and thus the daily early gathering is not achieved without some zeal, yet is appreciated all the more, perhaps, on that account.

### Probationers and Locality.

The theory that probationers should only be accepted from the town or county in which the hospital is situated, overlooks the impulse which urges people to go as far afield as possible for training or advice. The Scotch woman will come to England, the Londoner will go to Birmingham, the Irishwoman to Scotland. Inquiry shows that only as rare exceptions do inhabitants of provincial towns select their own hospital for their training school, and the reason may be that they believe higher esteem will be accorded to them among their friends if the brand of an unknown and presumably superior institution can be obtained. It has its advantages, for the complete change of outlook and habits which is involved in the process of being trained for a nurse is undoubtedly best carried out at a distance from anxious relatives, and when home ties can for the moment be completely severed.

### The Stock-pot.

It is quite certain that in hospitals where there is no stock-pot a great deal of valuable food finds its way into the pig-tub. From the moment when the cook begins her operations to the moment when the dinner is over, and the food which remains over is collected from the dishes, there are stages in which odds and ends of good victuals are liable to be wasted, unless a use can be found for them. The stock-pot should be of sufficient size to contain the leavings of two days, with an adequate proportion of water to cover its contents. It may be used as a receptacle for all the trimmings from the uncooked meat, including bone and fat. To this may with advantage be added pieces of gristle and skin left after carving diets, and lastly the well-chopped bones of certain joints which it is impossible when carving to free entirely from the meat. Also all carcasses of fowls, etc. On no account, however, should the leavings from the plates be made use of in this way, even when they have obviously not been touched. It is better to exclude all vegetables from the stock-pot, as they are fertile sources of fermentation or sourness. The pot should be kept constantly simmering, with a close lid, so that the loss by evaporation is kept under, and the liquor should be poured off every evening into a covered vessel, to be strained and skimmed the following morning. When mutton is bought in carcass, quite sufficient material for broth will be provided by the spare portions, unadapted for table use, without buying shin of beef. The best kind of stock pot is fitted with tap and strainer.

### District Nurses and Committees.

It is extremely unusual to hear of disagreements between private nurses and medical men, and such a thing is almost unheard of in institutions. For this reason the not uncommon instances which reach our ears of disputes arising in district work in the country gives rise to the surmise that there may be something unsatisfactory in the organisation under which the district nurse does her work. In several cases the fault can be traced directly to the interposition of the committee. However necessary a committee may be for raising funds and checking expenditure, its functions as intermediary between the doctor and nurse are a very doubtful advantage. Great discretion is needed. It is not always quite clear that this discretion is exercised. Many medical men dislike exceedingly the feeling that the conduct of all their cases among the poor is discussed at a weekly meeting of ladies empowered to inquire into every incident in the nurse's daily routine. The more interest the committee shows in the condition of the village invalids, the more mischief is likely to come of it. The causes why Mrs. A. was so slow in getting well of her confinement, and why old B. died suddenly when the doctor had omitted his usual visit, will make enough talk in a small village to cause the doctor to vow he will never have another nurse in the place. He knows his own business, and certainly is not called upon to explain his action to a group of ladies, however benevolent. He may be pardoned for feeling that the less they know about his cases the better; yet what nurse can withstand the influence of warm appreciation coupled with curiosity? Conceive the position of a parson, whose church workers held a weekly meeting for the purpose of interrogating the lay helper as to his daily doings. Must not friction be set stirring by such intervention, however kindly meant?

